# Supplementary figures and images for: Integrated analysis of miRNA landscape and cellular networking pathways in stage-specific prostate cancer
Source: PLoS One. 2019 Nov 22;14(11):e0224071. doi: 10.1371/journal.pone.0224071 (PMC6874298; doi:10.1371/journal.pone.0224071)

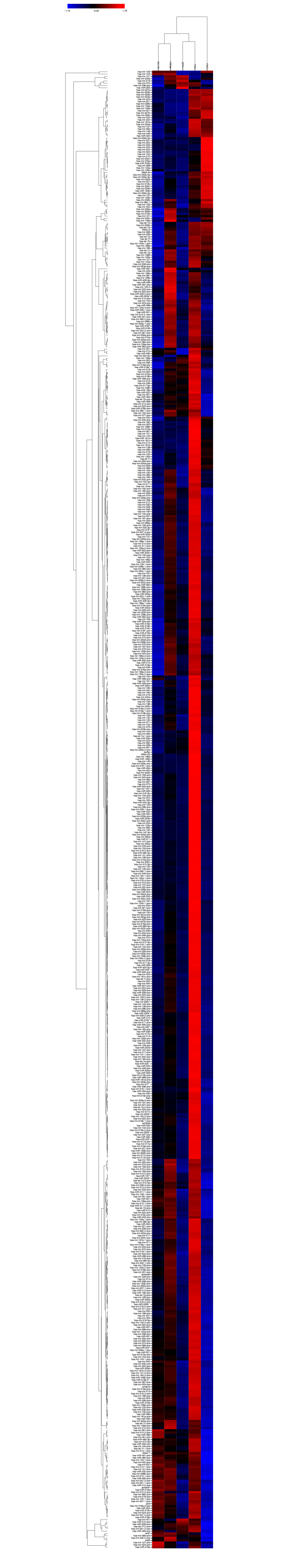

Supplement: S1 Fig — Expression of miRNAs differentially expressed and assessed in microarray analysis of RNA isolated from four different cell lines of prostate cancer (LNCaP, PC3, DU145, 22Rv1) compared with control cell line of prostate cancer (PrEc). The red color depicts high and green color showed a lower level of expression at p value <0.01. (TIF) [file pone.0224071.s001.tif]
