# Supplementary material for: Integrated analysis of miRNA landscape and cellular networking pathways in stage-specific prostate cancer
Source: PLoS One. 2019 Nov 22;14(11):e0224071. doi: 10.1371/journal.pone.0224071 (PMC6874298; doi:10.1371/journal.pone.0224071)
Supplement: S2 Fig — The internetworking relationship with miRNAs and plasma membrane protein P-glycoprotein (Pgp-plasma membrane protein). The miRNA-130a and miR-181a are downregulated in this pathway (green color) and linked with Pg and BCRP (breast cancer resistant protein). miR-133a and miR379 are involved in regulating the expression of MRP2. While miR-298, miR27a, miR331-5p and miR-130a are involved in regulating the expression of poly-glycoprotein (P-gp). Epithelial mesenchymal transition pathway. The miR-200b was downregulated during early-stage prostate cancer and was involved in inhibiting Jagged-2 (JAG2), one of the NOTCH ligands. Adipogenesis pathway. The expression of miR-326 was upregulated during the metastatic stage of prostate cancer, (red color) and involved in regulating the expression of CCAT/enhancer binding protein β (C/EBPβ), directly linked with the nuclear hormone receptor peroxisome proliferator-activated receptor-gamma (PPAR-γ). Bone metamorphosis signaling pathway. The expression of miR-140, miR-145, and miR-155 was upregulated, along with miR-140 and miR-145 were associated with modulation of gene SOX9, and miR-155 was involved in regulating the gene FOXO3A. Th1 pathway. In this pathway, the expression of miR-146a showed a higher level of expression (red color) and modulated the expression of NF-κB signaling. Th1 and Th2 pathway. In this pathway, the expression of miR-146a showed a lower level of expression (green color) and may modulate the expression of NF-κB signaling. (PDF) [file pone.0224071.s002.pdf]

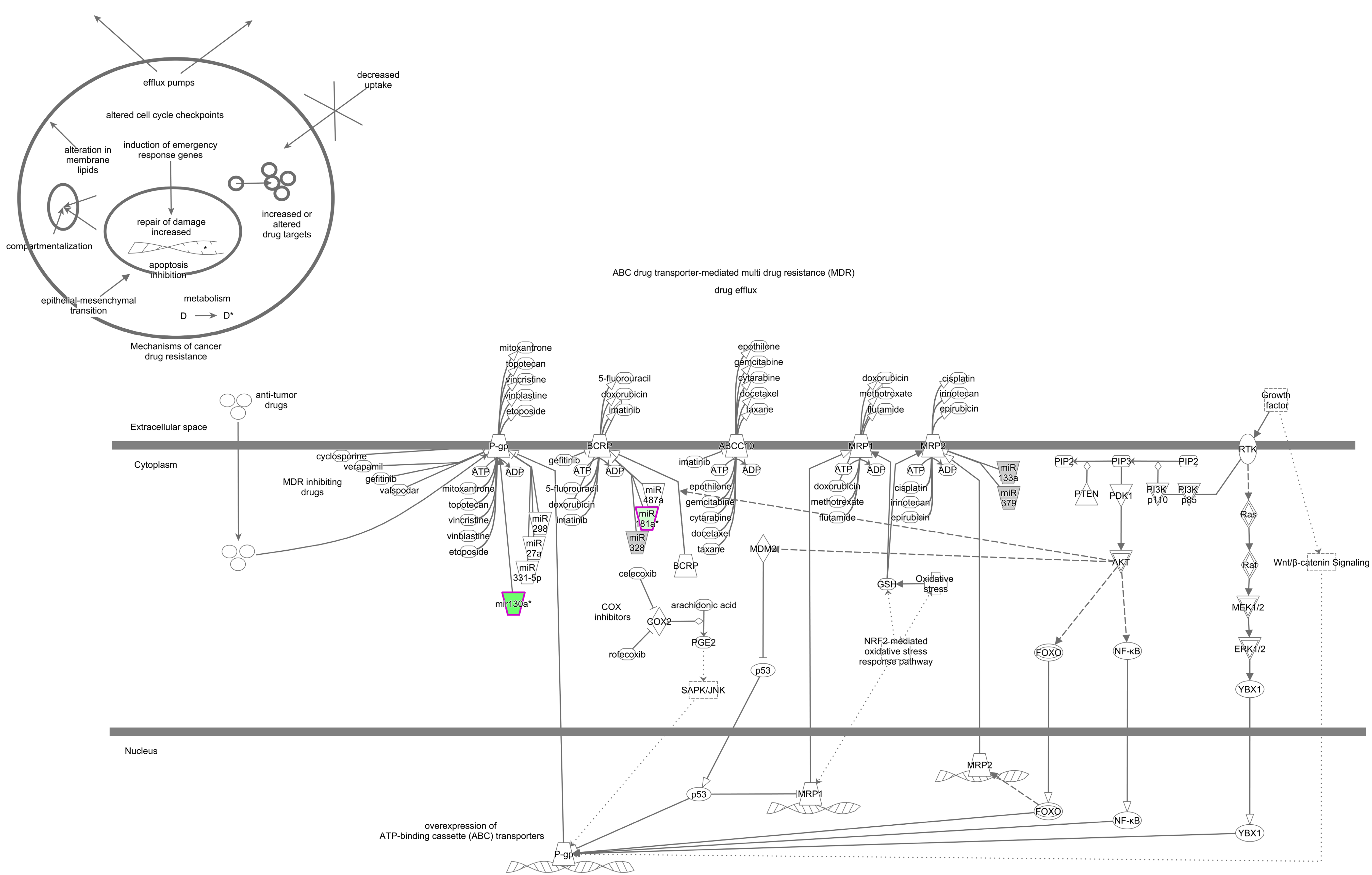

© 2000-2018 QIAGEN. All rights reserved.

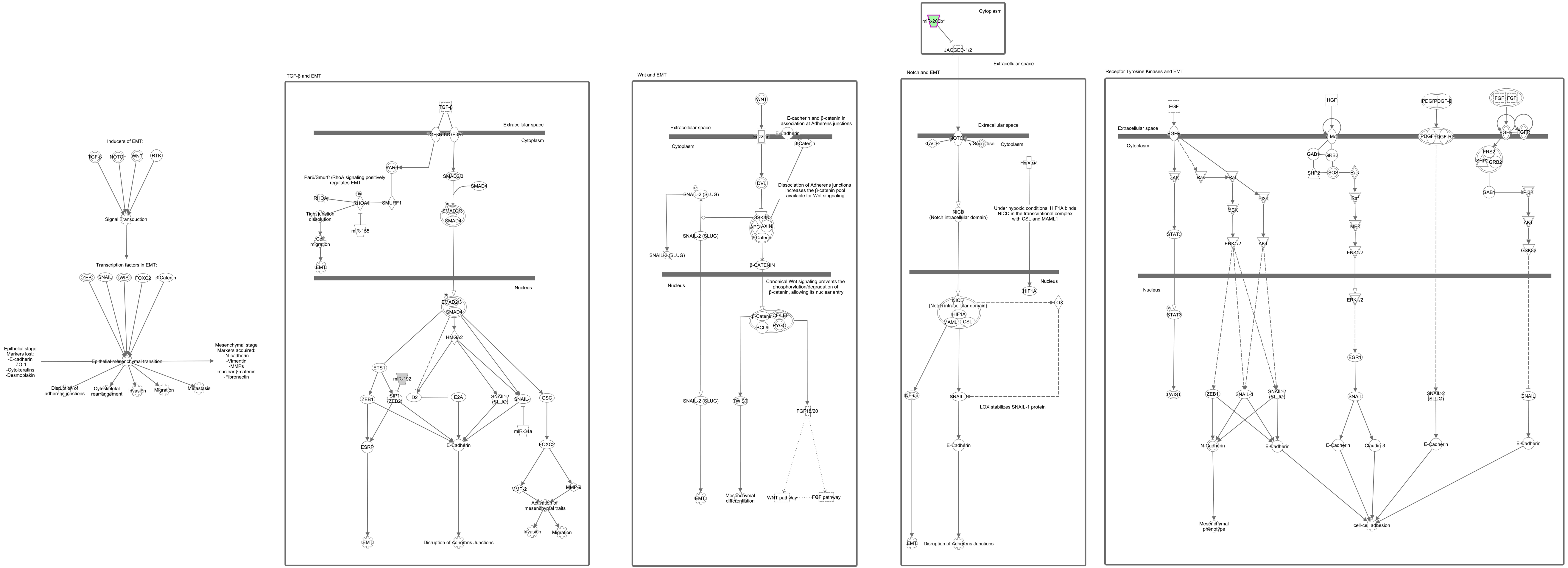

© 2000-2018 QIAGEN. All rights reserved.

Adipogenesis pathway

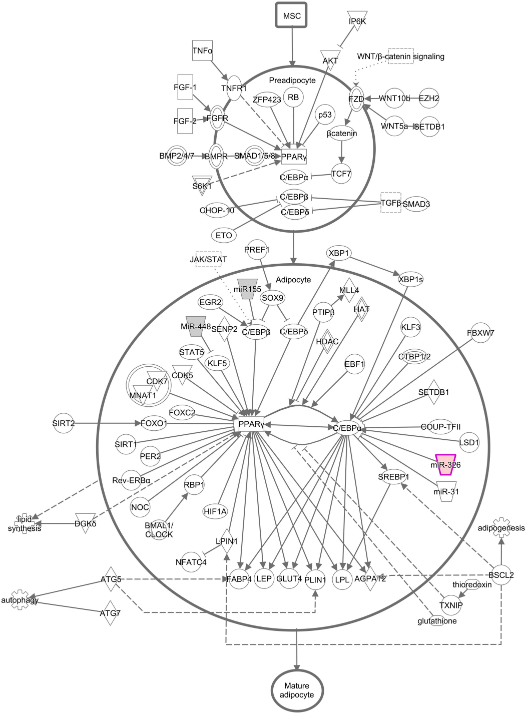

© 2000-2018 QIAGEN. All rights reserved.

Osteoarthritis Signalling Pathways

While other tissues of the joint and immune system are involved in osteoarthritis, the central cells are chondrocytes, which synthesize and reside in cartilage.

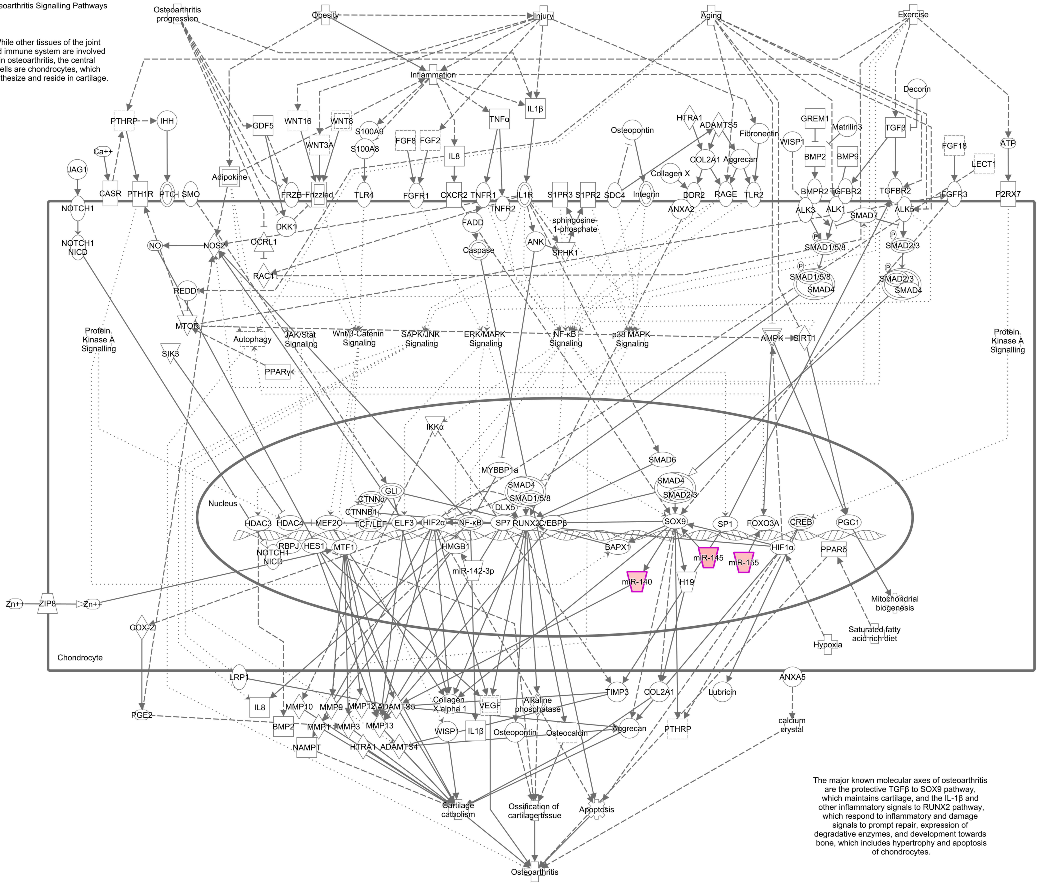

The major known molecular axes of osteoarthritis are the protective TGFβ to SOX9 pathway, which maintains cartilage, and the IL-1β and other inflammatory signals to RUNX2 pathway, which respond to inflammatory and damage signals to prompt repair, expression of degradative enzymes, and development towards bone, which includes hypertrophy and apoptosis of chondrocytes.

© 2000-2019 QIAGEN. All rights reserved.

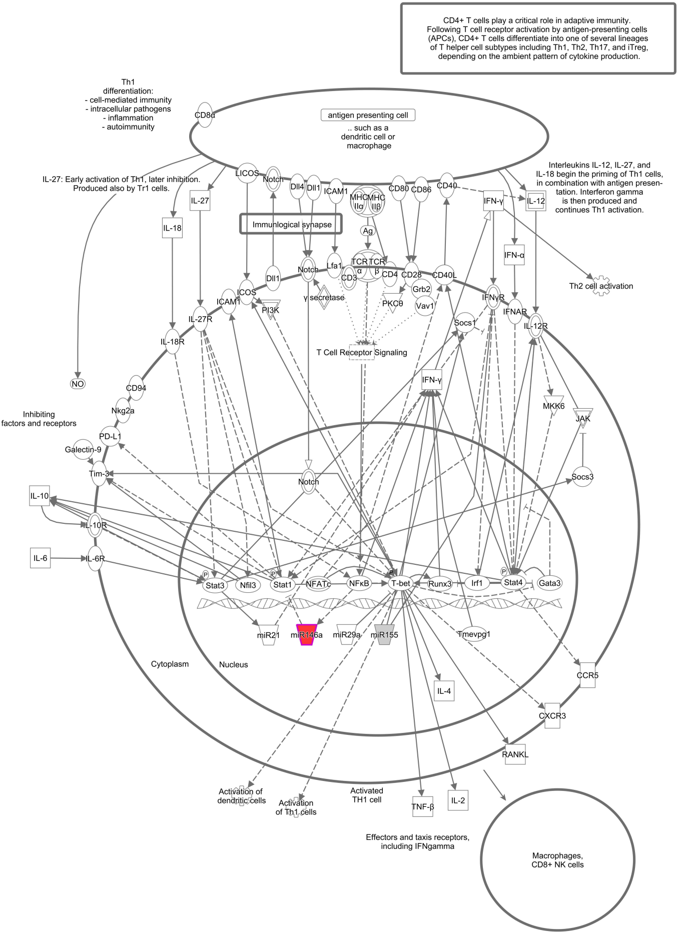

© 2000-2018 QIAGEN. All rights reserved.

Th1 and Th2 Activation Pathway : Incap : Expr Fold Change

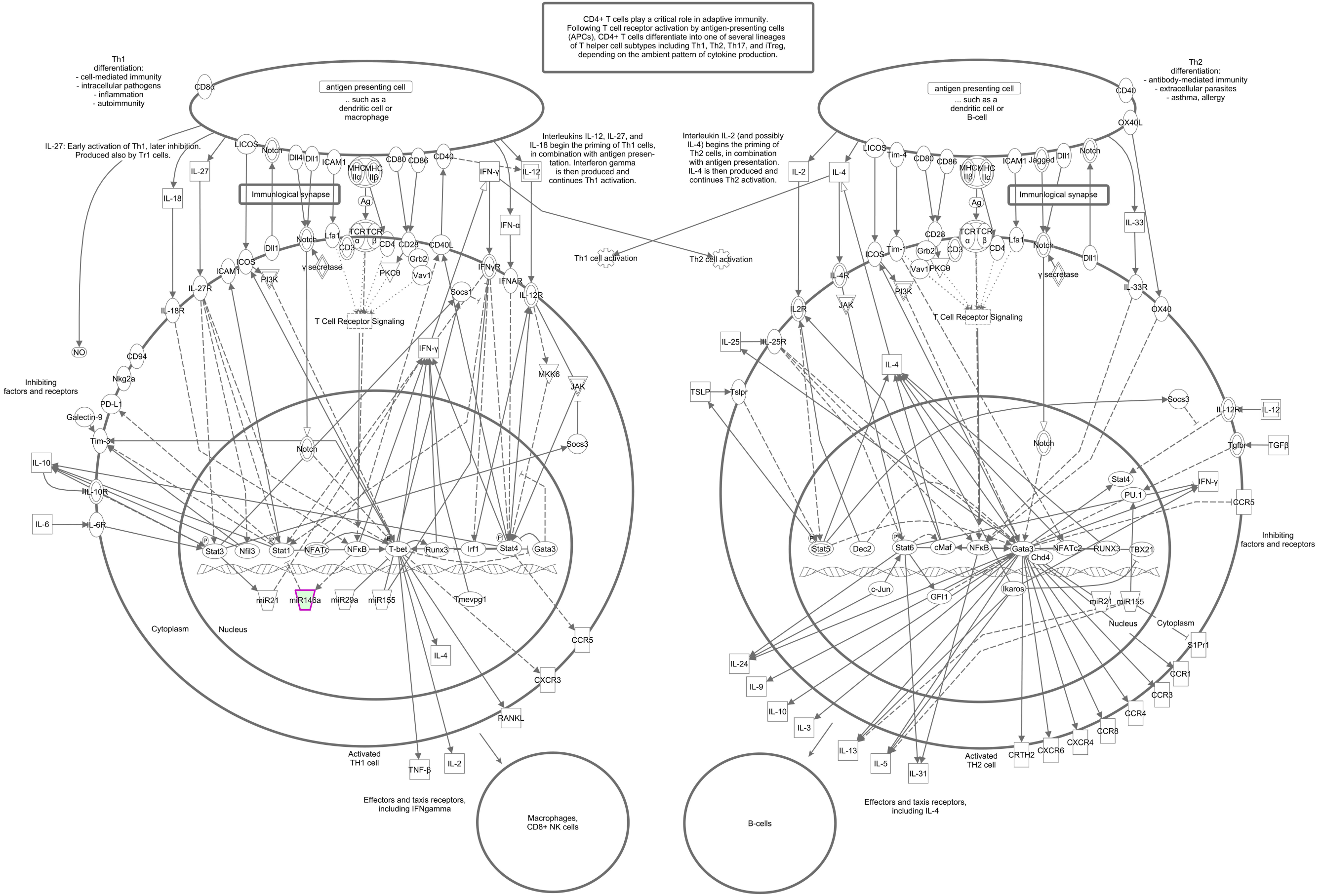

© 2000-2018 QIAGEN. All rights reserved.
